# Mapping the Structure and Dynamics of Genomics-Related MeSH Terms Complex Networks

**DOI:** 10.1371/journal.pone.0092639

**Published:** 2014-04-03

**Authors:** Jesús M. Siqueiros-García, Enrique Hernández-Lemus, Rodrigo García-Herrera, Andrea Robina-Galatas

**Affiliations:** 1 Ethical, Legal and Social Studies Department, National Institute of Genomic Medicine, Mexico City, D.F., Mexico; 2 Computational Genomics Department, National Institute of Genomic Medicine, Mexico City, D.F., Mexico; 3 Complexity in Systems Biology, Center for Complexity Sciences, National Autonomous University of Mexico, Mexico City, D.F., Mexico; Universidad de Zarazoga, Spain

## Abstract

It has been proposed that the history and evolution of scientific ideas may reflect certain aspects of the underlying socio-cognitive frameworks in which science itself is developing. Systematic analyses of the development of scientific knowledge may help us to construct models of the collective dynamics of science. Aiming at scientific rigor, these models should be built upon solid empirical evidence, analyzed with formal tools leading to ever-improving results that support the related conclusions. Along these lines we studied the dynamics and structure of the development of research in genomics as represented by the entire collection of genomics-related scientific papers contained in the PubMed database. The analyzed corpus consisted in more than 49,000 articles published in the years 1987 (first appeareance of the term *Genomics*) to 2011, categorized by means of the Medical Subheadings (MeSH) content-descriptors. Complex networks were built where two MeSH terms were connected if they are descriptors of the same article(s). The analysis of such networks revealed a complex structure and dynamics that to certain extent resembled small-world networks. The evolution of such networks in time reflected interesting phenomena in the historical development of genomic research, including what seems to be a phase-transition in a period marked by the completion of the first draft of the Human Genome Project. We also found that different disciplinary areas have different dynamic evolution patterns in their MeSH connectivity networks. In the case of areas related to science, changes in topology were somewhat fast while retaining a certain core-stucture, whereas in the humanities, the evolution was pretty slow and the structure resulted highly redundant and in the case of technology related issues, the evolution was very fast and the structure remained tree-like with almost no overlapping terms.

## Introduction

Complex networks theory is taking over where other *theoretical* approaches to complexity –such as synergetics, chaos theory and self-organized criticality– have had limited success. This is what Lászlo Barabási recently published in a paper called the *The Network Takeover* [1]. Complex networks theory has become the most recent attempt to tackle complexity, it has developed a great variety of methods and techniques with a wide scope of generality. So far, it has been possible to find properties such as power-law behavior in node degree distributions that are common among different kinds of systems, from regulatory transcription networks, to friendship networks, to epidemic networks [2]–[4]. But what seems to have been the difference between complex networks theory and the former approaches to complexity is its ability to incorporate (or build upon) empirical data: massive amounts of data. So, it has become an effective way to deal with the –so to speak– real complexity in which we are embedded, in a simple and beautiful way.

Science itself has been the subject of complex networks analysis [5]–[10]. Most studies are close to or framed inside scientometrics, the discipline that studies science by measuring and analysing its products. Network-based scientometrics rely on collaborations, coauthorship and citation networks. These kinds of networks are interesting for complex network analysis for reasons that can be found in current literature. Nevertheless, scientometrics has also produced many valuable findings that help us understand the sociology of science: the flows of knowledge, its cultural and disciplinary differences, as well as its political and economical aspects [11]–[13]. On those efforts we build upon. We are very much interested in how scientific knowledge is generated by a descentralized collectivity, and how it is organized. Our view is a little bit different from scientometrics in that we are mainly concerned on how to frame these data, patterns, processes and networks from the perspective of complex systems analysis, as an epistemological issue and as a subject of philosophy of science. We believe this is something that as far as we know, has not been systematically studied yet. For these matters, our methods are close to those of scientometrics, however, our interests are even closer to sociology and philosophy of science.

In this paper we explore the networks formed by the terms that describe the content of an article. These terms are known as MeSH, an acronym that stands for *Medical Subject Headings*. We are interested in the structure and dynamics of a network of the set of MeSH terms related to the term *Genomics*; we believe that to some extent, this particular MeSH terms network represents the image of a part of the biomedical human knowledge evolution and its current state in a specific time in history.

To be clear, MeSH is the controlled vocabulary defined by the National Library of Medicine of the United States that is used for indexing, cataloging, and searching for biomedical and health-related information and documents. It is composed of terms that name the descriptor of an object that can be a scientific paper, a film, a book or any other format in which medical knowledge is recorded and transmitted [14]. MeSH vocabulary constitutes what is called a MeSH tree, and it is structured alphabetically and hierarchically. By 2013, the tree included 17 hierarchies. Each hierarchy is a branch that goes from general to specific and every hierarchy is independent from the others, this means there is no dependance relationship between terms belonging to different hierarchies. For example: Hierarchy number 2, tagged [B], corresponds to *Organisms*; *Eukaryota* is the first subdivision of *Organisms*, that places *Eukaryota* in the classification system in *B01*. *Drosophila melanogaster* belongs to hierarchy [B01] but it goes all the way down to [B01.050.500.131.617.289.310.250.500]. The *Organisms* hierarchy is independent from the rest of hierarchies such as hierarchy [K], that makes reference to the *Humanities*.

From the 17 branches, 16 define the existing headings and 1 is a branch for all the existing subheadings to date. The 16 branches start with the root [A] that stands for the branch *Anatomy*, followed by [B] for *Organisms*, [C] being the root for *Diseases* and it goes all the way to the letter [N] that is for *Health Care*, then, it skips to letters [V] and [Z] for *Publication Characteristics* and *Geographicals* respectively. The remaining root is [Y] which includes all subheadings and is not part of the main MeSH tree. In order to define a precise meaning, a MeSH term starts with a heading and it is refined using subheadings. Every heading begins with uppercase letters and all subheadings begin in lowercase. When a composed MeSH term is formed, headings are followed by subheadings separated by slashes, for example: *Breast Neoplasms/diagnosis/genetics/psychology*.

MeSH is not a static vocabulary. The first list of terms was published in the 1950’s, and the first catalog of Medical Subject Headings appeared in the 1960’s. The 1960 MeSH edition included 4,400 descriptors and the second edition of 1963 got up to 5,700. The 2013 MeSH contains as many as 26,853 descriptors [15]. The MeSH catalog is updated every year and it is the work of a staff specialized in the subjects. The National Library of Medicine also receives recommendations regarding new terms and indexing. The whole enterprise is supported by many external professionals who are consulted for their expertise. The MeSH is used to index articles from the most important biomedical journals worldwide for the MEDLINE/PubMED database [14].

In this paper we explore the organization of knowledge that has been developed along with genomics research. Our networks display the emergence and decay of subjects, topics and disciplines as research in genomics has changed and we wanted to know how such behaviors have been taking place. Analyzing these networks we pretend to have a better understanding on how the topologies have changed during 25 years, how close are subjects from each other –what is the average shortest paths between nodes–, how different subjects and topics cluster, and how different areas of knowledge behave. Particularly we explore the structure and dynamics of different subnetworks defined by MeSH terms related to the areas of *science*, the *humanities* and *technology*.

The main findings in this work are that MeSH networks present a topology similar to that of small-world networks which is maintained along the years, independently of the rate of growth of such networks. Also we perceived differences in the connectivity patterns between different disciplines. Such patterns seem to be characteristic of each type of discipline. With regards to the dynamics of network growth we found evidence that seem to point out to the presence of three different regimes, roughly corresponding to the pre-Human Genome Project, the completion of the Human Genome Project (from now HGP) –whose behavior resembles a dynamic phase transition– and the post-genomic eras.

The rest of the manuscript is organized as follows, a section on Results and Discussion dealing with general aspects on MeSH networks topology, with network dynamics as well as a detailed analysis of some theme-specific subnetworks. Those networks have been chosen to represent different fields of inquiry. Representing a science-based theme are the subnetworks around the MeSH *Neoplasms*, for the humanities the networks chosen were encompassed by the MesH terms *Ethics* as well as *History*; and for the case of more technical/technological issues we chose the subnetworks related to the MeSH terms *Computational Biology* and *PCR*. After this discussion, a brief section dealing with the Materials and Methods used is included.

## Results and Discussion

### Networks Topology

We would like to report three main topological results. First, global networks (GNs) displayed an intricate and complex topology as can be seen in their respective network structure parameters [See Table S1]. Interstingly enough global networks always consist of only one connected component. This seems to imply that the whole corpus of biomedical knowledge related to genomic research is somehow integrated. This non-trivial structure may become evident by analyzing the values of quantities such as network density 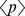 and clustering coefficient 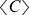. It becomes clear that the structure of such networks cannot be the result of random generated connections. In MeSH GNs, density decreased as the networks grow bigger (in 1991 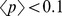 and since 2001 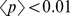). However 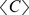 for all networks remained constant and high-valued for the whole history of genomics research (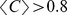). The topology of GNs, and specially their clustering coefficient values would be very unlikely in an Erdös-Rényi network with such density. It is also important to mention that 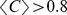 is indicative of non-tree-like networks.

The shortest average distance between any nodes *i, j* remained low for all GNs: 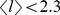. We believe such average distances would not be possible without a particular topology, such as in the Watts-Strogatz model, in which long distant links bring closer every node in the network that otherwise would be quite apart from each other. We also believe that there are communities that may lead to a small-world-like topology [16]. We may recall that Erdös-Rényi networks display low values of average shortest paths, and their clustering coefficient is also very low.

**Figure 1:**
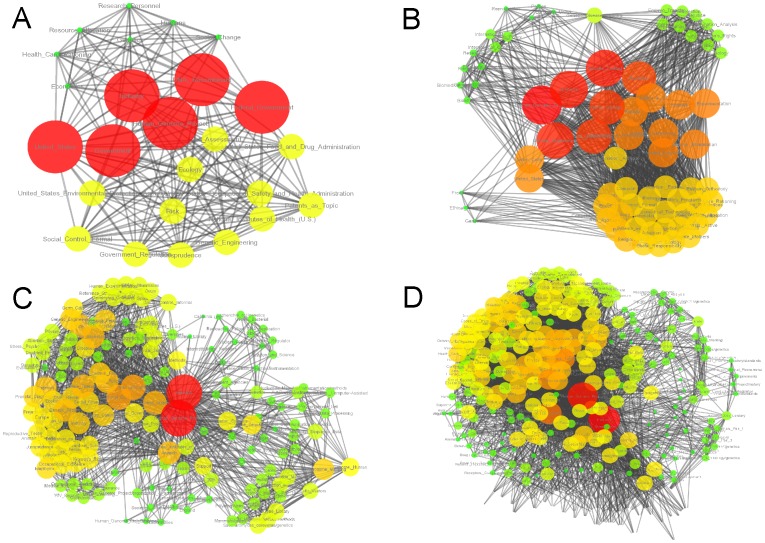
Global MeSH networks for the period (1987–1990). It is noticeable that there is a progressively growth of the network that induces greater variability in the connection patterns. New terms arise that lead to the generation of a more complex connectivity structure that reduces the relative importance of terms that were initially dominant. Nodes are size and color-coded according with their respective connectivity degree, i.e. big red nodes present a high connectivity, whereas small green nodes have lower values. This is an example of only four years.

**Figure 2:**
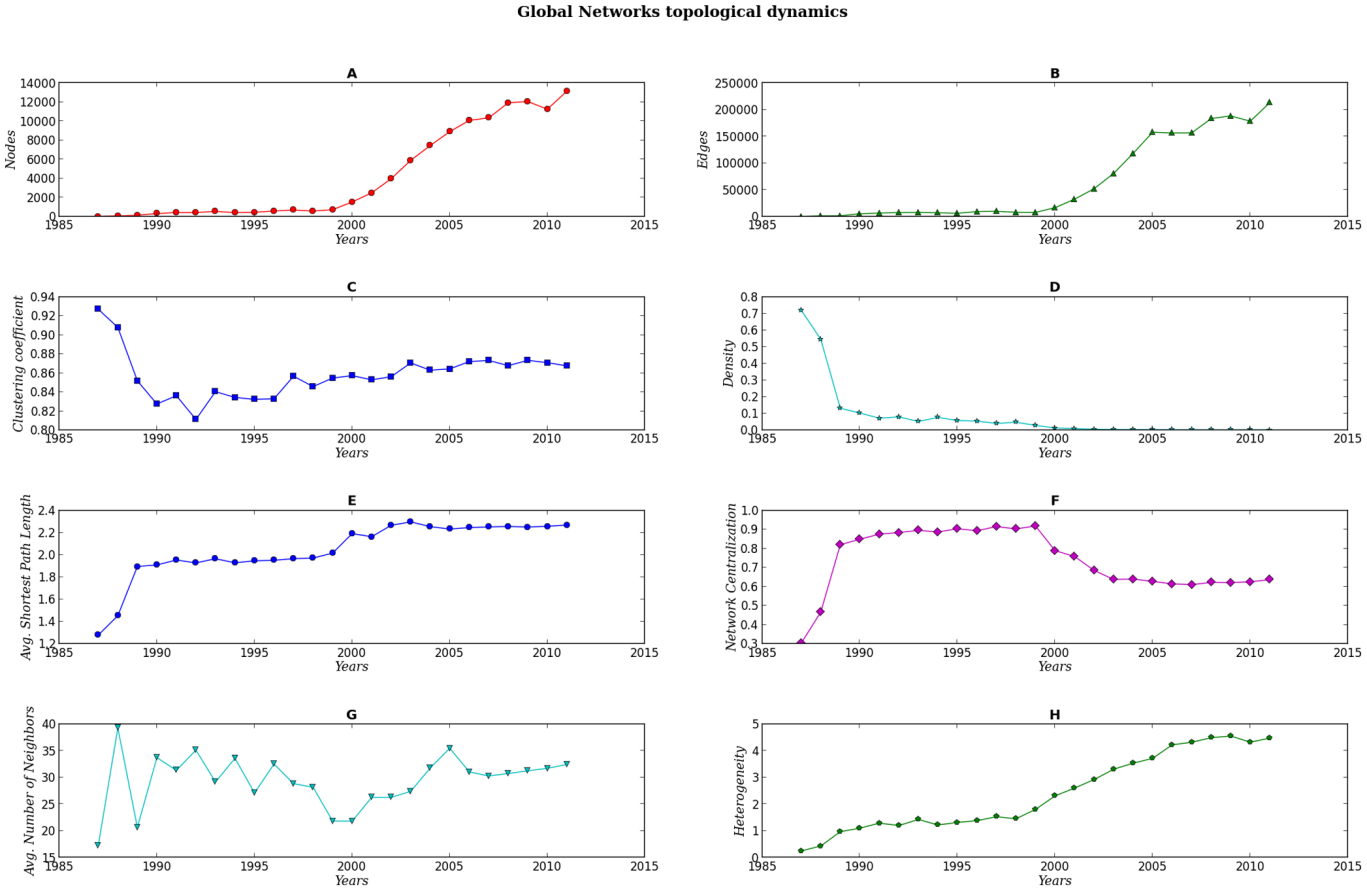
Dynamics of the topological parameters for the global networks. Panels A–H.

**Figure 3:**
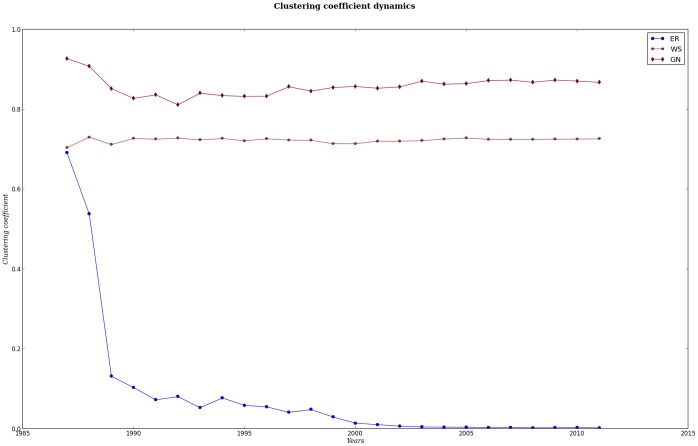
Average clustering coefficient versus time. We may see the average clustering coefficient for MeSH global (GN) networks, compared to random Erdös-Rényi (ER) and to small-world Watts-Strogatz (WS) networks with the same parameters as these. We can notice that MeSH global networks are not similar to tree-like networks (clustering coefficient = 0), neither to random networks (with clustering coefficient rapidly decaying) but seem to be close to small-world networks with large and persistent values of clustering. In the other hand, MeSH networks are also different from lattices since the average shortest path lenght values are not proportional to the size of the network but rather to the logaritm of such size.

All global networks displayed are sparse networks [Figures 1 and 2]. According to Watts and Strogatz, small-world topologies might be common to large, sparse or low density networks found in nature [17]. It has been pointed out by others that many real-world complex networks have a small-world effect, but they are different from a real small-world network in that their 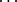 *average path length increases slower than any polynomial function of the system size* 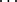 [16], [18]. If we look up at Figure 3 and Table S1 we may see that there is a strong resemblance of our GNs to small-world networks. Many of the small-world properties networks seem to be modular [16] and this also has implications for our work. If our GNs have a small-world effect topology then it means that genomics, as it has been growing, is becoming a vast sea that is quite navigable with islands of knowledge and lanes between them to be traveled. If GNs have such modular structure is something that remains to be elucidated and is part of our future agenda. Particularly, we would like to see if there are any emergent modules, how are they composed and how do they connect and affect each other. Most of all, emergent modularity might indicate non-trivial ways of organizing collectively produced knowledge, which would be interesting to be studied from a sociological, historical and philosophical point of view.

### Networks Dynamics

Following the parameters of the whole sequence of networks, from 1987 to 2011, it can be appreciated that they seem to grow in a structured manner. The first GN corresponds to 1987 (our initial time-point). During the first couple of years, the number of terms (nodes) in the network increased by addition of approximately ten new terms each year, starting with 25 nodes and 216 edges in 1987. From 1990 to 1999 the number of terms were added by hundreds and in the last decade it increased by thousands, so that by the year 2011, the number of terms summed up to 13,169 with 213655 edges. It is noteworthy that for the years that passed from the beginning of the Human Genome Project to the announcement of a working draft of the Human Genome in the year 2000, the networks seem to be limited in adding new terms. For instance, from 1990 to 1999 new terms were added at a rate of 58.2 per year, a very conservative number compared to a rate of 1035.8 terms per year from 2000 to 2011, in average. Specially interesting is the fact that in the year 2000 the curves for the number of nodes and edges start growing much faster than before. The sudden change in slope for the year 2000 curve suggests the beginning of a substantial increase in the exploration of genomic-related issues, contrary to the very limited scope before 2000 which might have been the result of the somehow narrow objective of sequencing the human genome in a limited time. It is in the year 2000 where there is the only significant change in the average shortest path length, from 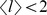 to 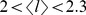.

From the 25 nodes in 1987, five nodes were the most connected ones. Among these, the term *Human Genome Project* was present in every GN. Another node that was one of the most connected terms over time is *Humans*, but in this first network it was not relevant (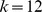). From 1989 to 1998 these two terms centralized the networks without rivals. In 1999 *Humans* had a higher degree of connectivity than *Human Genome Project*, and also, for the first time in ten years, a new term that will be important in the networks for the years to come emerges, such term was *Animals*. The year 2001 was the year in which the term *Genomics* became visible altogether with the term *Humans*, *Animals* and *Human Genome Project*. From this year onwards, this last term declined as an important, well-connected node, since its connectivity started to decrease reaching a 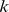 around the 300 in 2005, and remained close to this value for the following networks. Another moment that seems to be important in the history of genomics was the year 2003. During this year the term *Proteomics* gained in connectivity, comparable to the big nodes already mentioned. And for the year 2005, *Proteomics* along with *Proteomics, Methods*, were more connected than *Genomics* and it stayed like that for the following years. In our last network (*i. e.*, 2011), the most connected node was *Humans*, followed by *Animals*, *Proteomics*, and *Genomics*.

**Figure 4:**
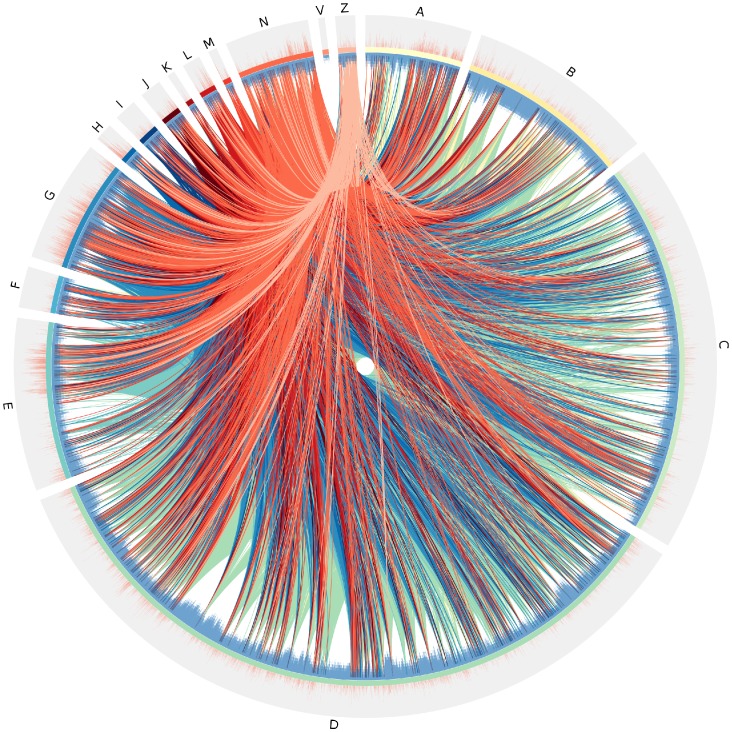
Circos plot of MeSH-tree headings. Circos plot displaying the interconnectivity among different headings belonging to genomics-related PubMed-indexed articles published in 2011. Letters correspond to the key in Table S2. The blue histogram in the inner circle represents the corresponding depth of MeSH tree levels for a given term. Higher bars are more specific terms. The orange histogram in the outer circle displays the base-10 logarithm of the number of published papers categorized for every MeSH term.

The complex connectivity structure within different MeSH terms lies behind the links among abstract terms, responsible for the conceptual coherence of the biomedical publication corpus represented in the PubMed database. In Figure 4 we can see a Circos plot [19] showing the interconnectivity between all different headings whose MeSH terms categorize PubMed-indexed publications related to *Genomics*, corresponding to the year 2011. Labels in every section correspond to the key in Table S2. We can notice that a blue histogram (shown in the inner circle) represents the corresponding depth of specific MeSH tree levels for a given term. Higher bars thus refer to more specific terms. The outer layer displays an orange histogram showing the base-10 logarithm of the number of published papers categorized for every MeSH term. Higher bars are then *hot topics*, generating a very large number of related publications.

As one can see in Figure 4, a multitude of different areas of knowledge converge to conform a highly multidisciplinary corpus in the research related to genomics. There is a dense, non-random interconnection pattern spanning across traditional fields of research (for instance, areas so-apparently disparate as pathology, history, molecular biology, sociology, and computer science are conceptually connected in this corpus). This may be indicative of current phenomena leading to an increase in adequacy and a reshape of the boundaries between contemporary research subjects in the evolution of afore-mentioned traditional fields of research. Very likely, this multidisciplinarity may be the driving force (or at least one of the forces) behind the establishment of new relations between already structured research areas, as well as the evolution of trending research topics. Further evidence in this regard may be observed when looking at the outer circle histograms in Figure 4 that present the number of articles published (on a logarithmic scale) that contain the connected MeSH terms. By looking at the blue histograms in the inner circle of Figure 4 we may notice that there is no apparent trivial relationship between the specificity of research topics (as represented by the number of hierarchical levels in the MeSH tree structure spanned by such topic) and the impact that such research may have in the overall biomedical community (as represented by the number of papers in the area given by the height of the orange histograms in the outer circle).

**Figure 5:**
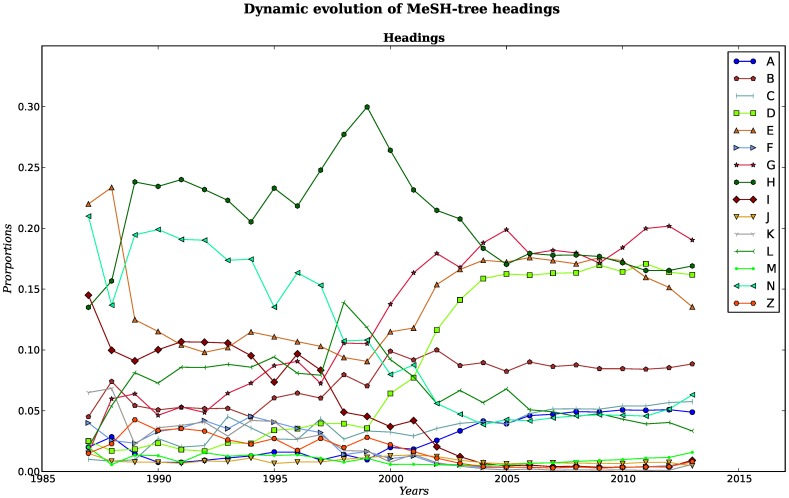
Dynamic evolution of MeSH-tree headings. Dynamics of the proportion of participation for every heading in terms of the number of PubMed indexed publications each year. There are noticeable changes in the trends for different issues that are consistent with different periods of time within the *genomics age*.

Since every heading belongs to a branch (or category), we also wanted to know the proportion of every branch in each GN by counting and plotting the normalized frequency of every root of each heading branch. The heading [V] never appeared in our networks, so we did not plot it. Semantic relationships between these terms are built and are behind the conceptual structure intrinsic to research in genomics. Figure 5 shows that in the first two-year period the proportions of headings get the positions that will keep for the following 7 years. This means that some headings abruptly increased while some others decreased their proportions in the 1987 and 1988 networks. From 1989 to 1995 most headings had a steady position. During this period the most represented headings were *H*, and *N*, the first one standing for *Disciplines and Occupations* and the second one standing for *Health care*. In the middle region (second period), one may find headings such as *L* for *Information science*, *E* for *Analytical, Diagnostic and Therapeutic Techniques and Equipment* and *I* for *Anthropology, Education, Sociology and Social Phenomena*. The least represented headings were *A* for *Anatomy*, *C* for *Diseases*, *D* for *Chemicals and Drugs*, *F* for *Psychiatry and Psychology*, *J* for *Technology, Industry, Agriculture*, *K* for *Humanities*, *M* for *Named Groups*, and *Z* for *Geographicals*.

From 1995 to 2004 things changed again: *H* and *L* went upwards and then downwards between 1996 to 1999. In 1999, the proportions of headings *E*, *D* and *G* grew rapidly. Contrary to these categories, category *I* abruptly started to decrease in 1997. The proportions for the remaining headings grew or diminished slightly. 2004 is the year when heading proportions stabilized and reached the positions that they have maintained since then. Headings *D*, *E*, *G*, *H* are placed at the top and moved all together as a group, although in 2009 *E*, *G* begun to split away. Heading *B* is alone, underneath the group of headings just mentioned. Under heading *B*, there is another well packed group of four headings. This group is composed by *A*, *C*, *L* and *N*. There is one last group of headings for which since 2004 their representation in the networks is close to zero, this group includes *F*, *I*, *J*, *K*, *M* and *Z*. It is noteworthy that *I* begun as an important heading during the first years, but its importance decreased, becoming almost unrepresented in later networks.

Figure 5 shows the dynamics in the proportion of branches (i.e. main categories in Table S2) for every GN. Analysis of this graph led us to suggest that there are three somewhat distinguishable periods in the evolution of genomics and related issues in the biomedical literature. The plot, as well as the corresponding networks, show that the benchmark for the identification of these periods is located between years 1999 and 2003. It is noteworthy that 2001 (the middle point in that interval) was the year that the draft of the sequence of the Human Genome was published in *Nature* and Celera’s paper on the methods used in the sequence draft was published in *Science* [20], [21]. Analysis of this graph in conjunction with the parameters obtained from the GNs lead us to hypothesize that in the period that ranged between 1999 and 2003, something similar to a *phase transition* took place. There were rearrangements in the proportions of each category that ended up in the actual configuration. Such transition may have been triggered by an important change in the networks parameters that occured during the 1999–2000 years, most surely due to the completion and publication of the first draft of the human genome. The number of nodes and edges increased substantially (from hundreds to thousands) and network centralization dropped from 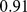 in 1999 to 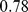 in 2000 and to 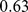 in 2003, due to the emergence of new terms, such as *Animals* and *Genomics* as highly centralized nodes. These two nodes rivaled with *Humans* and *Human Genome Project* as the two main hubs during the pre-genomic era. In summary, what we see in this apparent phase transition is an explosion in the variety of terms related to genomic research. So to speak, the number of nodes is at the same time, the number of different terms and different topics that might be seen as proxies for new grounds for genomics-framed exploration. In these years a new regime shown in Figure 2, came to be and its growth rate has been sustained so far. This must mean somehow that what sciences produce is sufficient at least to create the same or more diverse knowledge, a knowledge that is well connected to the major component and is well integrated as the clustering coefficient seems to suggest.

Our data about GNs also shows how in the postgenomic era, different headings (a proxy for general subjects) cluster together, and we assume that, at least in some cases, this is due to their conceptual affinity. For example we find that heading *H*, that stands for disciplines (in our networks mostly represented by genomics and proteomics) is highly correlated with the understanding of biological processes and chemicals, and with technology and methods (such as PCR) [see Figure 5]. It is interesting that the areas related to *Ethical, Legal and Social Issues of Genomics* (ELSI), lost a great part of their representation share once the Human Genome Project was accomplished. Since the graph shows proportions, it might be the case that the production of ELSI research is the same now as it was 20 years ago, but at the end the message is that it did not grow accordingly to the amount of subjects investigated, new areas of research, and new technologies –all this represented by the ever increasing size of the networks. ELSI research simply became less relevant once the project was over. Something similar happens to *Health care*, or heading *N*. Personalized, preventive and predictive medicine was the promise of genomics. All these important concepts were part of the vision of the proximal future in *Health care*. But once again, as other issues of public interest (or at least, closer to the public), its relevance in the share of proportions, faded away in the postgenomic era [Figure 5].

Along with the history of genetics and genomics [22], the dynamics of the proportions tells us a story of how genomics has departed from a set of preconceptions regarding human nature (as beings with rights and dignity) inherited from a bioethical and science policy and society agenda, in order to set the limits to human research. Nevertheless, the development of genomics has now created an image of what a living being is and how we fit into that description. It also tells us about what are the tools that have been applied in order to create that image. Such tools might be technological or conceptual. Furthermore, the very idea of genomics, reflected in the first networks and closely related to the HGP, must have changed considerably after 2003 and 2004. Today genomics may not be as much as the study of the whole genome but more of a generic name for a systemic view of the biology of living things. This drive, if there is such a thing, might be the focal point leading to a better understanding of how different levels of biological organization interact among each other, as well as to how different biological systems interact in an ecological fashion, as it is currently studied by metagenomics.

**Figure 6:**
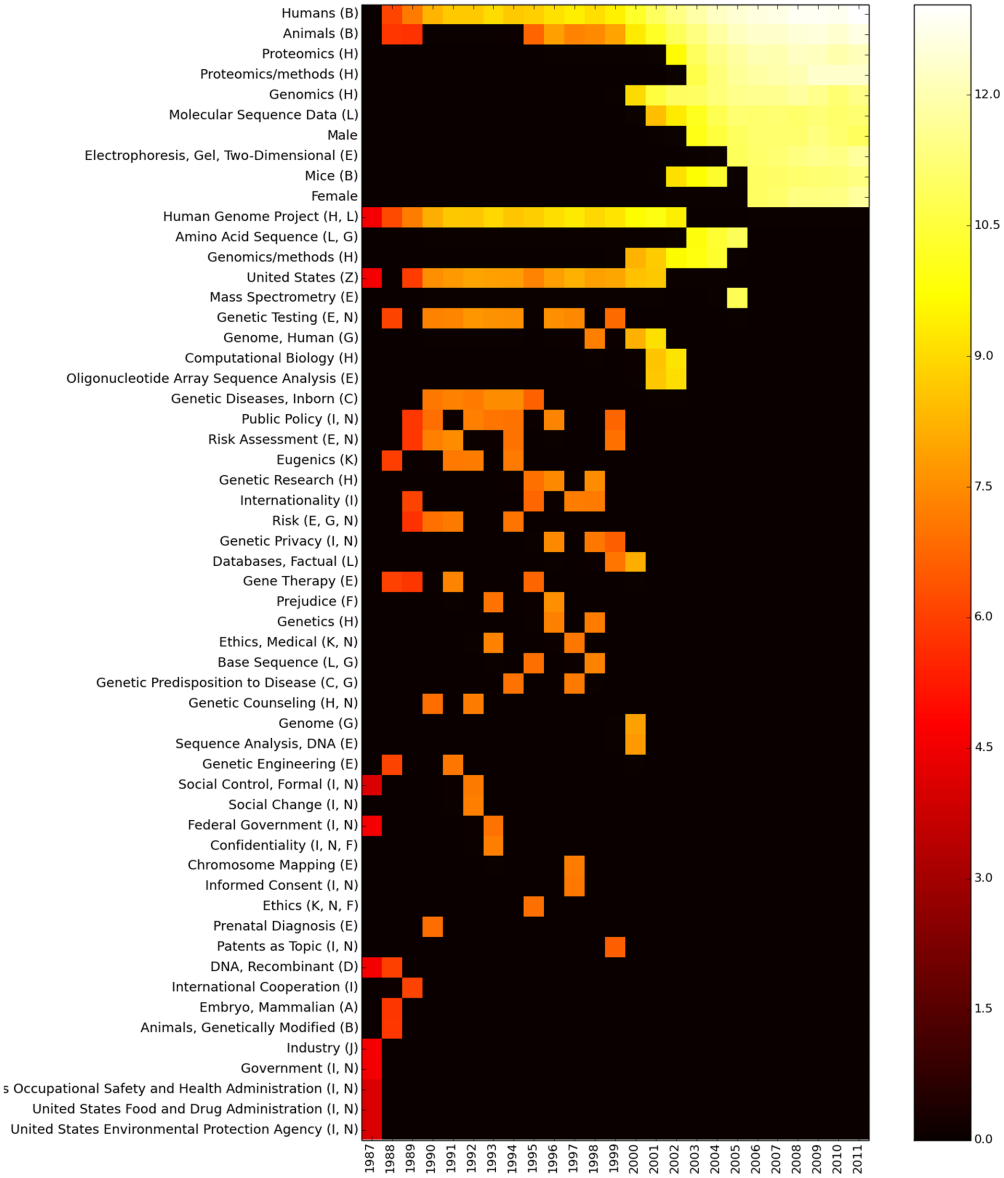
Dynamics of the Top-10 of the most connected MeSH terms. The Y-axis corresponds to a list of the 57 most connected MeSH terms in the global networks from 1987 to 2011. X-axis is the corresponding year. We may notice that some terms remain in the Top-10 list for several years, and even appear and dissappear from the list. Color-intensity is given by the 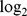 of the degree. The relative importance of the top-10 nodes reflects the fact that in the former years there was a lower number of potential connections in the global networks.

A closer analysis of the hot-topics was made by selecting the top-10 most connected MeSH terms for each year. It reveals interesting facts and trends while supporting our previous discussion. In figure 6 we can see that during the former years of genomic research (1987–2001) there were a lot of issues under discussion, dominated by health care and policy matters, as well as social phenomena, in what may be called an ELSI stage of genomics. In the other hand, most recent years show a completely different trend by having fewer issues, most of them related to more technical and scientific aspects of genome research.

By examining the color code in figure 6 (which corresponds with the log-2 of the degree of each node) we can see that the discussion has become more rich in recent years with a much larger number of edges for the Top-10 topics that in those corresponding to the former years.

### Subnetworks

Since the GNs include the whole set of MeSH terms related to the term *Genomics* we were able to substract smaller networks based on particular terms such as *Neoplasms*, *Ethics*, *History*, *Computational biology*, and *Polymerase Chain Reaction* (commonly known as *PCR*). The networks reflect the use of the specific term in such a context for every year explored. Some terms like *Ethics* appeared in the earliest networks (1988), some others like *Computational biology* come to be part of the GN in the year of 1996. We chose these subjects because we were very much interested in the approach of different “disciplines” or perspectives towards a particular topic (in our case that topic is genomics), how they behave over the years and if in that regard there are substantial differences between the humanities (*e.g. Ethics and History*), science (*e.g. Neoplasms* research) and technological development (*e.g. Computational biology and PCR*). For these set of SNs we recorded values for the number of nodes 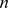 and edges 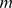, clustering coefficient 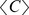, network centralization 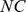, shortest path length 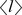 and density 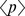 [see Tables S3–S7].

**Figure 7:**
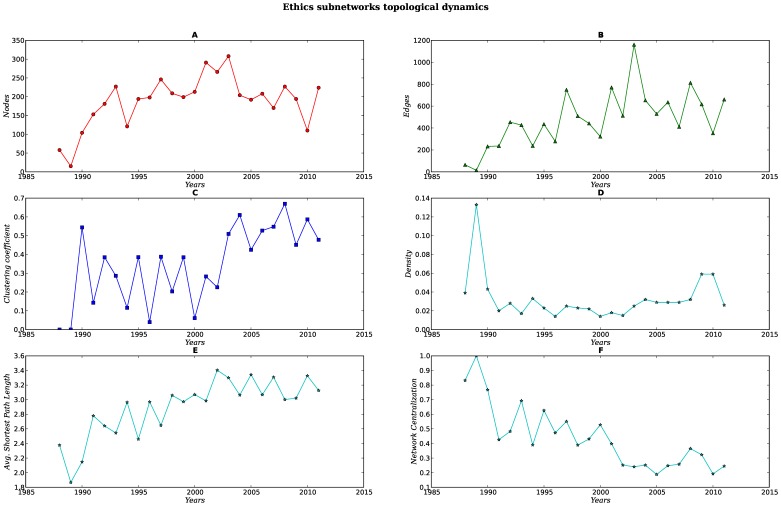
Dynamics of the topological parameters for the Ethics networks. Panels A–F.

**Figure 8:**
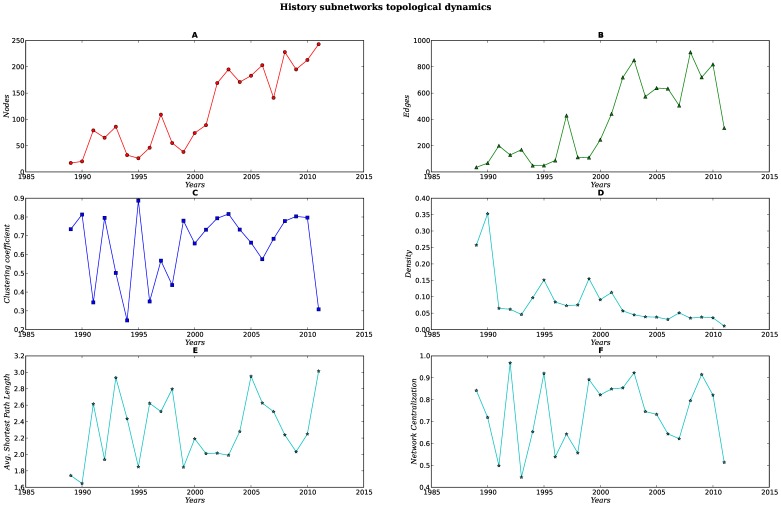
Dynamics of the topological parameters for the History subnetworks. Panels A–F.

**Figure 9:**
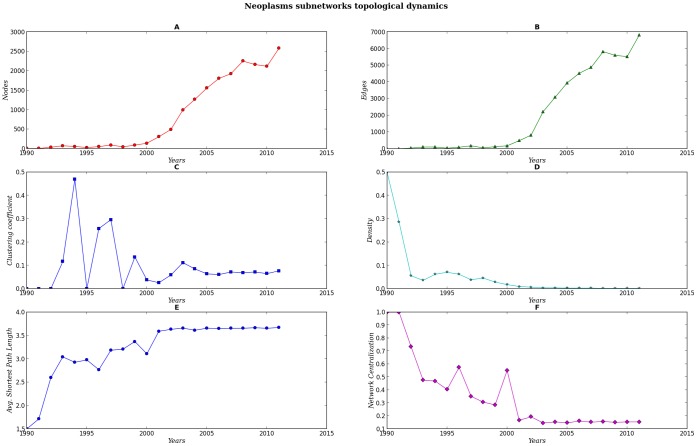
Dynamics of the topological parameters for the Neoplasms networks Panels A–F.

**Figure 10:**
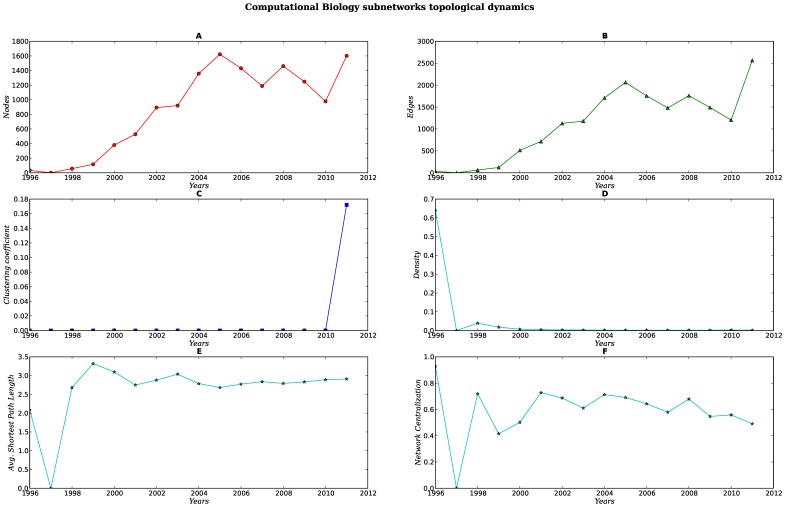
Dynamics of the topological parameters for the Computational Biology subnetworks. Panels A–F.

**Figure 11:**
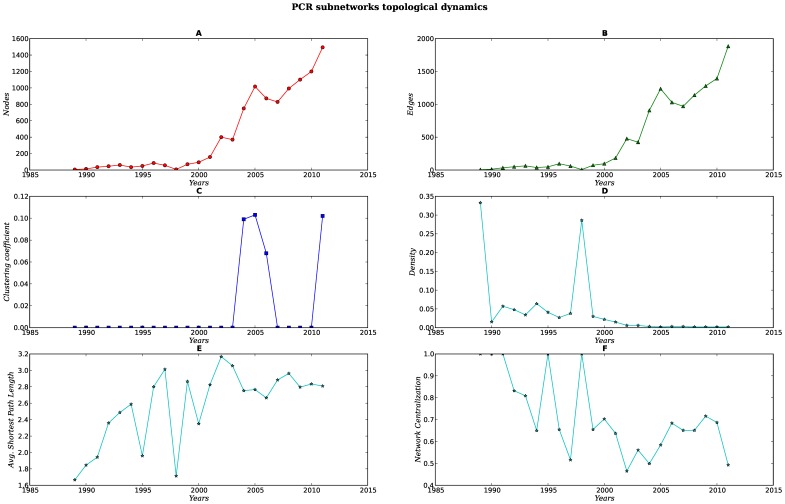
Dynamics of the topological parameters for the PCR subnetworks. Panels A–F.

Every set of subnetworks (SNs) display a different structure and dynamics [see Figures 7, 8, 9, 10, 11]. When compared against each other, we noticed that despite the low density of each network, there were important differences in the clustering coefficient values. While the set of networks for the humanities had high clustering coefficient values [Figures 7, 8 Panel C], the science subnetwork (represented by the SN *Neoplasms*), were slightly higher than those presented by a random network [Figure 9 Panel C]. Contrary to the results of the humanities and science SNs clustering coefficients, the technology SNs were below the results for a random network [Figures 10, 11 Panel C]. We believe that these results somehow mirror the nature of the different areas. The results of these thematic SNs suggest that not all areas of inquiry behave in the same way. The humanities appear to move at a slower pace as compared to the other areas. The humanities seem to be quite redundant in their subjects and concepts. For instance, in the case of *Ethics*, concepts that are central to debates are words like *justice*, *dignity*, *equity*, words that have been in the ethics vocabulary for hundreds of years. Interestingly enough, in an article recently published in the *New York Times*, Nicholas Christakis makes reference to an apparent state of stagnation in the Social Sciences [23]. From what we see in our *Ethics* and *History* SNs, it seems that what is to be for the Social Sciences it might be also true for the Humanities –although the pupose of the formers is different from the latters, since the Social Sciences purport themselves as *sciences*. We also explored the content of the triangles (responsible for the clustering coefficient) of a fraction of all SNs for each of these subjects, nevertheless, we were able to see that for *History*, a network with very high clustering coefficient and network centralization [see Tables S3–S7], the terms with the highest connectivity were the terms present as two of the nodes in the triangle. Quite different from this, the *Ethics* SNs had a high clustering coefficient and network centralization, still triangles were not dominated by highly connected nodes, on the contrary, clusters were more diverse.

In the case of science, we think that the low but constant clustering is a sign of the fact that in science there must be some conserved knowledge as a scaffold or dynamic supportive structure on which novelty and new ideas are built upon. We wonder what is the nature of such conserved knowledge and we will try to identify it as part of our future work. However we think that perhaps these scaffolds rest on the form of models, and model organisms. Finally, technology seems to be an area that is always *on a rush*. These SNs have average clustering coefficients 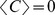 for most of the years. We suggest that this is the case because technology, although built on previous technologies, it might not need to make reference to them because, possibly, there are different technical solutions to the same problem. Even if this can be the most common case, we found that for the *PCR* case there are some years in which there was a clustering coefficient over zero. In these *PCR* networks, one of the nodes in the triangle was the new technology, the other the precedent technology and another node that could change but probably was relevant for the new technology, such as gene expression and in a lesser degree, sequencing, proteomics, methods, and organisms. In summary, the humanities change very slowly compared to science and technology. Science develops fast but needs to rely on previous knowledge, and technology always goes ahead, almost with no explicit reference to previous works. In order to test the validity of these ideas and as part of our future work, we will perform studies to properly characterize these areas as communities or neighborhoods and study their local dynamics compared to those of the GNs [16], [24].

In the networks we studied, there were terms that were used both as headings and subheadings. The terms *Neoplasms*, *Computational Biology* and *Polymerase Chain Reaction* exist in the MeSH database as headings only, which means that they only appear at the beginning of the MeSH term. The terms *Ethics* and *History* exist as headings and subheadings, that is, they can be at the beginning or in the middle of a MeSH term. Headings and subheadings can be distinguished from each other because headings always start with a capital letter and subheadings always begin in lowercase, *e. g.*, the term *Ethics*, can be found as **Ethics** or as **ethics**, and can be the heading of a MeSH term such as: *Ethics, Medical*, or as a subheading like: *Informed Consent/ethics/legislation & jurisprudence*. We believe that this is interesting because ethics was an important area of research that was promoted since the beginning of genomics and the Human Genome Project. We think that the fact that ethics moved from a heading into a subheading as years went by, is telling us that there is a *cultural* progressive shift regarding the place of moral values in science. At the beginning, the concept of ethics was about a discipline and linked necessarily to the Human Genome Project and as things were developing, ethics became part of many other, more specific areas related to genomics, biomedical research and its clinical and societal consequences. Ethics changed from being the big word for socially legitimating the Human Genome Project to the ethics of many things. Still, it is important to mention that even if the results regarding the shift from ethics as a heading to ethics as a subheading might suggest some ideas related to the role of moral values and the dynamics of science, we recognize that due to the lack of more evidence (that we will explore in the future) we cannot generalize any of these results.

It is no news to say that the Human Genome Project was primarily a State project with the Department of Energy and the National Institutes of Health of the United States as the main actors behind one of the biggest scientific, technological and economical enterprises in the course of research history. It is evident that genomics research somehow was born with the HGP. Even if the HGP was launched officially in 1990, there is a record of publications regarding it since 1987. Such early publications clearly reflect the nature of the project. All articles published from 1987 to 1989 are by no means technical in terms of science but technical regarding what was needed economically, socially and politically for the proper development of the HGP. Some of the terms that appear in the first three years networks were *United States*, *Risk Assessment*, *Resources Allocation*, *Government Regulation*, and *Ecology*, as well as others of more ethical concern, like *Eugenics*, *Abortion*, and *Christianity*.

This is interesting in several ways: it is so for the history of science, technology, and society studies because the HGP is probably one of the first big scientific projects that it was planned ahead and looked for a formal way to gain social legitimacy; this strategy was an antecedent for another big project, that of Nanotechnology. It was not only planned in terms of budget, it somehow implied political planning (in order to get the budget approved), but it also looked for social approval. It is no coincidence that James Watson –being the head of the National Center for Human Genome Research– suggested (even before the HGP was formally launched) to include a research area for the study of the Ethical, Social and Legal implications of Genomics.

From the history of the HGP and the networks we built, it seems that genomics was the outcome of a *top-down* style of planning science. Genomics might have appeard in the long run as a consequence of technological and scientific interests as it has happened to many other scientific areas. For example, the Sanger sequencer was first developed in 1977, which means that there were some antecedents heading towards the emergence of genomics as a discipline, and of course, it played an important part in its future appearence. But it was in the context of the HGP that genomics really fluorished. In the particular case of genomics, we think that the HGP played a central role in accelerating the process. The behavior we just described for the first 10 years of the HGP might have be the reflection of a centralized, *top-down*, control over the development of the project, with a very precise objective in mind. Once the draft of the Human Genome was released and published, –results along with the technology developed in the former years– opened the door to a *bottom-up* style science, in which scientific communities were relatively free to set their particular problems and follow their own interests. Such change might be related to the change in the rate of new terms added per year, which somehow can be seen as a proxy of new emerging research subjects and areas.

Finally, it is also interesting the fact that methodologies like those of complex network theory can be useful for historians. Data mining and complex networks visualization and analysis can be a way for supporting ideas and intuitions with data, regarding how and why science changes and how does it interact with technology and society. In this regard, the whole set of terms related to the Ethical, Legal and Social issues (from now on ELSI) found in the first 10 years of the HGP is the most informative corpus of data on the social perceptions, conceptions and concerns about the human genome. The human genome as the blueprint of life, as the deepest repository of our identity as individuals and as a species. What is even more interesting is that the HGP shaped and materialized such concerns that were slowly framed over the 20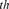 century as has been noted by other authors [25].

At the end, *what are our networks?* MeSH terms that form the networks are a simplified representation of the content of scientific papers. Scientific papers are the result of many people’s work, a work that goes beyond authors. A paper is the outcome of many individuals, such as the peers that review the proposal in order to get funding, research ethics committees, and the paper reviewers as well (just to mention a few). It is also part of an institutional enterprise. So to speak, every paper is a piece of knowledge that is socially agreed (at least in terms of the community that believe that the work should be published). In our specific case, the pieces of knowledge are not only technical and scientific in their nature, but also include knowledge from the humanities and the social sciences. The content of every paper includes directly or indirectly a diversity of topics, from a diversity of sources. By this, we do not imply that, for example, to be part of a paper, ethical issues need to have a MeSH term associated to the subject Ethics in it. What we mean is that there are papers that in order to be published, need many people to have agreed upon its content, including those of the ethics committee that had to approve and follow up the project. Therefore, our networks are the concise description of these pieces of knowledge interacting massively among each other. They are the image and the collective evolution of a world-wide community around *Genomics*, arguing and agreeing on *what* and *how* ideas are or may become *knowledge*. At the end, this is the substance of our networks [10] and the main object of study in this work.

A plethora of questions arise from these studies. The implications of the highly structured connectivity patterns in these networks for the evolution of scientific knowledge (or at least for the case of biomedical research) are taunting and yet to be discovered. Also intriguing is whether such structure emerges from the particular classification approach in the case of MeSH terms or is interwoven in other, more general approaches to knowledge classification, perhaps even with ontological and epistemological implications.

There is more work to be done. In particular, there are some issues that we want to address in the near future. Some of these issues are related to community-identification and how these communicate among each other; what are the implications of a small-world topology for knowledge organization, and how the diversity of terms and edges impacts the topology and dynamics of the networks. We would also like to study in more detail, from a mathematical as well as from a historical point of view, the suggested phase transition.

Finally, quantitative studies in the evolution of knowledge are now arising. These studies may help us gain in understanding of the structure and evolution of ideas behind academic publications, not only (as is the present case) in the biomedical sciences, but in every documented field of human inquiry. Computational studies, as well as data and text mining techniques are now being supplemented with analysis and visualization tools that will allow researchers in this nascent discipline to construct more insightful models of cognition in a wide variety of fields. These changes may lead to an eventual development of data-driven approaches to epistemological studies of science.

## Materials and Methods

We extracted the MeSH terms that describe all the articles indexed in PUBMED that included the MeSH term *Genomics*. Data-mining was done using custom scripts written in Python. To create and analyze the networks we used Cytoscape 3.0.0 [26] and NetworkX [27], a Python library for the study of complex networks.

We searched for all the articles that included the MeSH term *Genomics* to December 2011, and retrieved their information in the MEDLINE format, as it is offered by the PUBMED webpage. We implemented BioPython’s API in order to parse our MEDLINE files and generate a connectivity map in which source and target nodes are the MeSH terms that describe the articles including the MeSH term *Genomics* and the link between the nodes are the PUBMED IDs, or PMIDs.

Major topics, or the main topic of an article is a MeSH term marked with an asterisk. Since we were not interested at the moment in separating major from minor topics, we eliminated the asterisk from the terms in the connectivity map. Once the connectivity map was clean, we created a connectivity map for every year, starting from 1987 and ending in 2011. To every connectivity map a bash script was run to count and give order to the pairs of MeSH terms. By doing this we replaced the PMID that linked every pair of terms with a “weight” according to the number of occurrences of that pair. For example if a pair of terms like *animals* and *humans* comes to occur 100 times, then, the weight of that pair is 100. For this paper we did not analyze the role of the weight of edges in the structure and dynamics of the networks, but that will be part of our future work.

We used Cytoscape 3.0.0 to generate the MeSH terms network for every year; we named these networks *global networks* or GN. For each year we counted the frequencies of every main category heading [see Table S2], that is, recorded how many *A’s*, *B’s*, *C’s* an so forth. The results were normalized and plotted using Python. We also counted the number of levels for each MeSH term from the table distributed by the National Library of Medicine. Given this, we encoded them to the Circos plot histogram format (blue histogram) 4. For the orange histogram, for each term we counted the number of articles containing it for every year from our data base and encoded it in the Circos plot histogram format 4. For the links connecting terms in the central area of the Circos plot, we used the information already obtained for our networks. In order to do this, we encoded the information in the Circos plot link format 4.

We identified the nodes and edges that are in the intersection of the global networks (GN) for the first network in 1987, to the 1990, 2001 and 2011 GNs. The intersections helped us to identify a small set of nodes such as: *Humans, Animals, Genomics, Human Genome Project, Risk* in order to analyze the dynamics of their degree, clustering, centrality, betweenness centrality and specially, to see who were their neighbors along this history of genomics throughout the years. For this purpose we used NetworkX. By using Cytoscape 3.0.0 [28], we identified the most centralized terms and the emergence of new central terms at different moments of history.

We extracted a group of subnetworks, for terms that are related to different areas of knowledge that have become part of genomic research. These areas are the study of *Neoplasms*, for a scientific object of research, for the humanities we generated a subnetwork for *Ethics* and *History*, and finally, for the technological areas we did it for *Computational biology* and *Polymerase Chain Reaction*. In order to extract these SNs, we searched the connectivity map of each year and chose every pair of nodes that included the term of interest. In the case of *Computational biology* we looked for the root *comput*, for the others we searched for the whole word, that is *Neoplasms*, *Ethics*, *History* and *Polymerase Chain Reaction*. We also extracted the triangles of the *Polymerase Chain Reaction*, and *Computational Biology* SNs to those few years with a clustering coefficient above zero; we also extracted the triangles to the *History* SNs because we wanted to see what was behind the high network centralization and clustering coefficient. Subnetworks were extracted using a bash script and Cytoscape 3.0.0 was used to visualize and analyze them; triangles were extracted with a bash script as well.

## Supporting Information

Table S1Data for the set of MeSH terms **Global Networks** for 25 years. First column contains the year, second column (n) is the number of nodes, third column (m) is the number of edges, fourth column (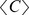) is the average clustering coefficient, fifth column (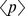) is the networks’ density, sixth column (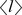) is the shortest average path length, and seventh column (NC) is the network centralization.(PDF)

Table S2 MeSH terms are organized according to a hierarchical multilayered structure of main categories, subcategories and so on. This table displays the two principal layers and the issues spanned by them. Additional layers provide specificity to the conceptual ontology.
(PDF)

Table S3Data for the MeSH term **Neoplasms** networks for 22 years. First column contains the year, second column (n) is the number of nodes, third column (m) is the number of edges, fourth column (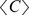) is the average clustering coefficient, fifth column (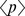) is the networks’ density, sixth column (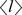) is the shortest average path length, and seventh column (NC) is the network centralization.(PDF)

Table S4Data for the MeSH term **Ethics** networks for 24 years. First column contains the year, second column (n) is the number of nodes, third column (m) is the number of edges, fourth column (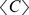) is the average clustering coefficient, fifth column (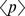) is the networks’ density, sixth column (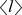) is the shortest average path length, and seventh column (NC) is the network centralization.(PDF)

Table S5Data for the MeSH term **History** networks for 23 years. First column contains the year, second column (n) is the number of nodes, third column (m) is the number of edges, fourth column (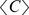) is the average clustering coefficient, fifth column (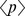) is the networks’ density, sixth column (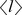) is the shortest average path length, and seventh column (NC) is the network centralization.(PDF)

Table S6Data for the of MeSH term **Computational Biology** networks for 16 years. First column contains the year, second column (n) is the number of nodes, third column (m) is the number of edges, fourth column (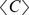) is the average clustering coefficient, fifth column (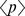) is the networks’ density, sixth column (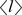) is the shortest average path length, and seventh column (NC) is the network centralization.(PDF)

Table S7Data for the MeSH term **Polymerase Chain Reac-tion** networks for 23 years. First column contains the year, second column (n) is the number of nodes, third column (m) is the number of edges, fourth column (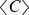) is the average clustering coefficient, fifth column (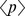) is the networks’ density, sixth column (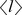) is the shortest average path length, and seventh column (NC) is the network centralization.(PDF)
