# Supplementary material for: Mapping the Structure and Dynamics of Genomics-Related MeSH Terms Complex Networks
Source: PLoS One. 2014 Apr 3;9(4):e92639. doi: 10.1371/journal.pone.0092639 (PMC3974714; doi:10.1371/journal.pone.0092639)
Supplement: Table S1 — Data for the set of MeSH terms Global Networks for 25 years. First column contains the year, second column (n) is the number of nodes, third column (m) is the number of edges, fourth column () is the average clustering coefficient, fifth column () is the networks’ density, sixth column () is the shortest average path length, and seventh column (NC) is the network centralization. (PDF) [file pone.0092639.s001.pdf]

Table S1. Data for the set of MeSH terms **Global Networks** for 25 years.

**Years**	**n**	**m**	**⟨C⟩**	**⟨p⟩**	**⟨l⟩**	**NC**
1987	25	216	0.9269565217	0.72	1.28	0.3043478261
1988	73	1434	0.9078624823	0.5456621005	1.4543378995	0.4671361502
1989	157	1621	0.8519214556	0.1323697534	1.8961293484	0.820388751
1990	326	5497	0.8276320734	0.1037659273	1.9102973101	0.8491358025
1991	432	6770	0.8363086488	0.0727206325	1.9547563805	0.8756488426
1992	436	7659	0.8113732076	0.0807655805	1.92860909	0.8842099687
1993	551	8037	0.840577467	0.0530407524	1.9661771985	0.8956648452
1994	434	7291	0.8344765407	0.0775960239	1.9295452369	0.8872316312
1995	462	6265	0.8324841669	0.0588312627	1.9472068062	0.9038668301
1996	591	9612	0.8331138521	0.0551320657	1.9524907511	0.8936548589
1997	700	10097	0.8567102662	0.0412712038	1.9667974658	0.915565011
1998	583	8213	0.8458654495	0.0484105792	1.9719014695	0.9031412838
1999	739	8075	0.8547908002	0.0296122718	2.0168175701	0.9186734473
2000	1541	16824	0.8573402846	0.0141786831	2.1919372646	0.7894433052
2001	2496	32798	0.8529629404	0.010533246	2.1638209111	0.7588122516
2002	3983	52341	0.8562280134	0.006600251	2.2665661053	0.6856074011
2003	5911	81045	0.8706930057	0.004639891	2.2972871907	0.6382156446
2004	7455	118502	0.8630025109	0.0042649961	2.2546963205	0.6405271802
2005	8917	158218	0.864404664	0.0039801282	2.2328671076	0.6278340853
2006	10094	156690	0.8721232881	0.0030760097	2.2460428104	0.6151970778
2007	10359	156922	0.8732427216	0.0029249611	2.2520855786	0.6111146326
2008	11955	183833	0.8678093861	0.0025728488	2.256171661	0.62292872
2009	12068	188600	0.873405057	0.0025902225	2.2511955195	0.6215286931
2010	11269	178752	0.8708983352	0.0028154556	2.2576028028	0.6258898753
2011	13169	213655	0.8676337479	0.0024641687	2.2689740703	0.6375172208

Global Networks. n:nodes, m: edges, $\langle C \rangle$: Clustering coefficient, $\langle p \rangle$: density, $\langle l \rangle$: Shortest average path length,NC: Network centralization
