# Supplementary material for: Mapping the Structure and Dynamics of Genomics-Related MeSH Terms Complex Networks
Source: PLoS One. 2014 Apr 3;9(4):e92639. doi: 10.1371/journal.pone.0092639 (PMC3974714; doi:10.1371/journal.pone.0092639)
Supplement: Table S2 — MeSH terms are organized according to a hierarchical multilayered structure of main categories, subcategories and so on. This table displays the two principal layers and the issues spanned by them. Additional layers provide specificity to the conceptual ontology. (PDF) [file pone.0092639.s002.pdf]

Table S2. MeSH Tree. MeSH terms are organized according to a hierarchical multilayered structure of main categories, subcategories and so on. This table displays the two principal layers and the issues spanned by them. Additional layers provide specificity to the conceptual ontology.

Main Category	Subcategory Key	Subcategories
A Anatomy	A01	Body regions
	A02	Musculoskeletal system
	A03	Digestive system
	A04	Respiratory system
	A05	Urogenital system
	A06	Endocrine system
	A07	Cardiovascular system
	A08	Nervous system
	A09	Sense Organs
	A10	Tissues
	A11	Cells
	A12	Fluids and Secretions
	A13	Animal Structures
	A14	Stomatognathic System
	A15	Hemic and Immune Systems
	A16	Embryonic Structures
	A17	Integumentary System
	A18	Plant Structures
	A19	Fungal Structures
	A20	Bacterial Structures
	A21	Viral Structures
B Organisms	B01	Eukaryota
	B02	Archaea
	B03	Bacteria
	B04	Viruses
	B05	Organism Forms
	C01	Bacterial Infections and Mycoses
	C02	Virus Diseases
	C03	Parasitic Diseases
	C04	Neoplasms
	C05	Musculoskeletal Diseases
	C06	Digestive System Diseases

Continued on next page

Table S2 – **Continued from previous page**

Main Category	Subcategory Key	Subcategory Key
C<br>Diseases	C07	Stomatognathic Diseases
	C08	Respiratory Tract Diseases
	C09	Otorhinolaryngologic Diseases
	C10	Nervous System Diseases
	C11	Eye Diseases
	C12	Male Urogenital Diseases
	C13	Female Urogenital Diseases and Pregnancy Complications
	C14	Cardiovascular Diseases
	C15	Hemic and Lymphatic Diseases
	C16	Congenital, Hereditary, and Neonatal Diseases and Abnormalities
	C17	Skin and Connective Tissue Diseases
	C18	Nutritional and Metabolic Diseases
	C19	Endocrine System Diseases
	C20	Immune System Diseases
	C21	Disorders of Environmental Origin
	C22	Animal Diseases
	C23	Pathological Conditions, Signs and Symptoms
	C24	Occupational Diseases
	C25	Substance-Related Disorders
	C26	Wounds and Injuries
D<br>Chemicals and Drugs	D01	Inorganic Chemicals
	D02	Organic Chemicals
	D03	Heterocyclic Compounds
	D04	Polycyclic Compounds
	D05	Macromolecular Substances
	D06	Hormones, Hormone Substitutes, and Hormone Antagonists
	D08	Enzymes and Coenzymes
	D09	Carbohydrates
	D10	Lipids
	D12	Amino Acids, Peptides, and Proteins
	D13	Nucleic Acids, Nucleotides, and Nucleosides
	D20	Complex Mixtures
	D23	Biological Factors
	D25	Biomedical and Dental Materials

Continued on next page

Table S2 – **Continued from previous page**

**Main Category**	**Subcategory Key**	**Subcategory Key**
	D26	Pharmaceutical Preparations
	D27	Chemical Actions and Uses
E<br>Analytical,Diagnostic and<br>Therapeutic Techniques and Equipment	E01	Diagnosis
	E02	Therapeutics
	E03	Anesthesia and Analgesia
	E04	Surgical Procedures, Operative
	E05	Investigative Techniques
	E06	Dentistry
	E07	Equipment and Supplies
F<br>Psychiatry and Psychology	F01	Behavior and Behavior Mechanisms
	F02	Psychological Phenomena and Processes
	F03	Mental Disorders
	F04	Behavioral Disciplines and Activities
G<br>Phenomena and Processes	G01	Physical Phenomena
	G02	Chemical Phenomena
	G03	Metabolic Phenomena
	G04	Cell Physiological Phenomena
	G05	Genetic Phenomena
	G06	Microbiological Phenomena
	G07	Physiological Phenomena
	G08	Reproductive and Urinary Physiological Phenomena
	G09	Circulatory and Respiratory Physiological Phenomena
	G10	Digestive System and Oral Physiological Phenomena
	G11	Musculoskeletal and Neural Physiological Phenomena
	G12	Immune System Phenomena
	G13	Integumentary System Physiological Phenomena
	G14	Ocular Physiological Phenomena
	G15	Plant Physiological Phenomena
	G16	Biological Phenomena
	G17	Mathematical Concepts
H<br>Disciplines and Occupations	H01	Natural Science Disciplines
	H02	Health Occupations
I<br>Anthropology,Education,	I01	Social Sciences
	I02	Education

Continued on next page

Table S2 – **Continued from previous page**

**Main Category**	**Subcategory Key**	**Subcategory Key**
Sociology and Social Phenomena	I03	Human Activities
J<br>Technology,Industry,Agriculture	J01<br>J02	Technology, Industry, and Agriculture<br>Food and Beverages
K<br>Humanities	K01	Humanities
L<br>Information Science	L01	Information Science
M<br>Named groups	M01	Persons
N<br>Health care	N01<br>N02<br>N03<br>N04<br>N05<br>N06	Population Characteristics<br>Health Care Facilities, Manpower, and Services<br>Health Care Economics and Organizations<br>Health Services Administration<br>Health Care Quality, Access, and Evaluation<br>Environment and Public Health
V<br>Publication Characteristics	V01<br>V02<br>V03<br>V04	Publication Components<br>Publication Formats<br>Study Characteristics<br>Support of Research
Z Geographicals	Z01	Geographic Locations
