# Supplementary material for: Mapping the Structure and Dynamics of Genomics-Related MeSH Terms Complex Networks
Source: PLoS One. 2014 Apr 3;9(4):e92639. doi: 10.1371/journal.pone.0092639 (PMC3974714; doi:10.1371/journal.pone.0092639)
Supplement: Table S3 — Data for the MeSH term Neoplasms networks for 22 years. First column contains the year, second column (n) is the number of nodes, third column (m) is the number of edges, fourth column () is the average clustering coefficient, fifth column () is the networks’ density, sixth column () is the shortest average path length, and seventh column (NC) is the network centralization. (PDF) [file pone.0092639.s003.pdf]

Table S3. Data for the MeSH term **Neoplasms** networks for 22 years.

**Years**	**n**	**m**	**⟨C⟩**	**⟨p⟩**	**⟨l⟩**	**NC**
1990	4	3	0	0.5	1.5	1
1991	7	6	0	0.286	1.714	1
1992	37	37	0	0.056	2.595	0.734
1993	75	101	0.117	0.036	3.042	0.476
1994	58	102	0.469	0.062	2.926	0.518
1995	30	31	0	0.071	2.977	0.404
1996	53	86	0.258	0.062	2.768	0.575
1997	95	175	0.296	0.039	3.185	0.351
1998	48	52	0	0.046	3.207	0.307
1999	92	121	0.136	0.029	3.371	0.285
2000	140	174	0.038	0.018	3.108	0.551
2001	307	479	0.026	0.01	3.591	0.167
2002	494	815	0.059	0.007	3.632	0.193
2003	997	2223	0.112	0.004	3.659	0.146
2004	1267	3087	0.085	0.004	3.612	0.154
2005	1558	3945	0.064	0.003	3.657	0.147
2006	1805	4519	0.061	0.003	3.648	0.16
2007	1927	4878	0.072	0.003	3.654	0.152
2008	2251	5821	0.069	0.002	3.655	0.157
2009	2163	5593	0.072	0.002	3.667	0.151
2010	2117	5525	0.065	0.002	3.654	0.154
2011	2580	6808	0.077	0.002	3.672	0.154

Neoplasms. n:nodes, m: edges, $\langle C \rangle$: Clustering coefficient, $\langle p \rangle$: density, $\langle l \rangle$: Shortest average path length,NC: Network centralization
