# Supplementary material for: Mapping the Structure and Dynamics of Genomics-Related MeSH Terms Complex Networks
Source: PLoS One. 2014 Apr 3;9(4):e92639. doi: 10.1371/journal.pone.0092639 (PMC3974714; doi:10.1371/journal.pone.0092639)
Supplement: Table S4 — Data for the MeSH term Ethics networks for 24 years. First column contains the year, second column (n) is the number of nodes, third column (m) is the number of edges, fourth column () is the average clustering coefficient, fifth column () is the networks’ density, sixth column () is the shortest average path length, and seventh column (NC) is the network centralization. (PDF) [file pone.0092639.s004.pdf]

Table S4. Data for the MeSH term **Ethics** networks for 24 years.

**Years**	**n**	**m**	$\langle \mathbf{C} \rangle$	$\langle \mathbf{p} \rangle$	$\langle \mathbf{l} \rangle$	**NC**
1988	58	64	0	0.039	2.377	0.832
1989	15	14	0	0.133	1.867	1
1990	104	232	0.544	0.043	2.147	0.768
1991	153	236	0.143	0.02	2.78	0.426
1992	181	453	0.385	0.028	2.641	0.483
1993	227	427	0.286	0.017	2.544	0.693
1994	121	237	0.116	0.033	2.964	0.39
1995	194	435	0.386	0.023	2.459	0.626
1996	198	278	0.04	0.014	2.97	0.473
1997	246	749	0.388	0.025	2.648	0.551
1998	209	509	0.203	0.023	3.06	0.389
1999	199	442	0.385	0.022	2.972	0.431
2000	213	322	0.061	0.014	3.07	0.528
2001	291	770	0.283	0.018	2.984	0.398
2002	266	512	0.225	0.015	3.404	0.252
2003	308	1161	0.509	0.025	3.3	0.241
2004	204	653	0.61	0.032	3.063	0.252
2005	192	529	0.425	0.029	3.343	0.188
2006	208	635	0.527	0.029	3.068	0.248
2007	170	411	0.548	0.029	3.308	0.258
2008	227	813	0.67	0.032	3.002	0.365
2009	194	617	0.451	0.059	3.021	0.323
2010	110	352	0.587	0.059	3.328	0.192
2011	224	661	0.478	0.026	3.126	0.245

Ethics. n:nodes, m: edges, $\langle C \rangle$: Clustering coefficient, $\langle p \rangle$: density, $\langle l \rangle$: Shortest average path length,NC: Network centralization
