# Supplementary material for: Mapping the Structure and Dynamics of Genomics-Related MeSH Terms Complex Networks
Source: PLoS One. 2014 Apr 3;9(4):e92639. doi: 10.1371/journal.pone.0092639 (PMC3974714; doi:10.1371/journal.pone.0092639)
Supplement: Table S5 — Data for the MeSH term History networks for 23 years. First column contains the year, second column (n) is the number of nodes, third column (m) is the number of edges, fourth column () is the average clustering coefficient, fifth column () is the networks’ density, sixth column () is the shortest average path length, and seventh column (NC) is the network centralization. (PDF) [file pone.0092639.s005.pdf]

Table S5. Data for the MeSH term **History** networks for 23 years.

**Years**	**n**	**m**	$\langle \mathbf{C} \rangle$	$\langle \mathbf{p} \rangle$	$\langle \mathbf{l} \rangle$	**NC**
1989	17	35	0.735	0.257	1.743	0.842
1990	20	67	0.813	0.353	1.647	0.719
1991	79	199	0.345	0.065	2.615	0.499
1992	65	129	0.795	0.062	1.938	0.968
1993	86	169	0.502	0.046	2.935	0.446
1994	32	48	0.249	0.097	2.435	0.654
1995	26	49	0.887	0.151	1.849	0.92
1996	46	87	0.35	0.084	2.623	0.539
1997	109	429	0.567	0.073	2.523	0.643
1998	55	111	0.437	0.075	2.797	0.557
1999	38	109	0.78	0.155	1.845	0.892
2000	74	245	0.659	0.091	2.191	0.822
2001	89	442	0.732	0.113	2.011	0.849
2002	169	719	0.793	0.057	2.016	0.854
2003	195	850	0.816	0.045	1.99	0.923
2004	171	573	0.733	0.039	2.28	0.746
2005	183	638	0.663	0.038	2.951	0.733
2006	203	634	0.575	0.031	2.626	0.644
2007	141	505	0.684	0.051	2.521	0.622
2008	228	911	0.778	0.035	2.238	0.796
2009	195	720	0.803	0.038	2.033	0.915
2010	213	818	0.797	0.036	2.249	0.821
2011	243	334	0.308	0.011	3.015	0.514

History. n:nodes, m: edges, $\langle C \rangle$: Clustering coefficient, $\langle p \rangle$: density, $\langle l \rangle$: Shortest average path length,NC: Network centralization
