# Supplementary material for: Mapping the Structure and Dynamics of Genomics-Related MeSH Terms Complex Networks
Source: PLoS One. 2014 Apr 3;9(4):e92639. doi: 10.1371/journal.pone.0092639 (PMC3974714; doi:10.1371/journal.pone.0092639)
Supplement: Table S6 — Data for the of MeSH term Computational Biology networks for 16 years. First column contains the year, second column (n) is the number of nodes, third column (m) is the number of edges, fourth column () is the average clustering coefficient, fifth column () is the networks’ density, sixth column () is the shortest average path length, and seventh column (NC) is the network centralization. (PDF) [file pone.0092639.s006.pdf]

Table S6. Data for the MeSH term **Computational Biology** networks for 16 years.

**Years**	**n**	**m**	**⟨C⟩**	**⟨p⟩**	**⟨l⟩**	**NC**
1996	33	34	0	0.64	2.091	0.929
1997	0	0	0	0	0	0
1998	57	62	0	0.039	2.684	0.719
1999	116	122	0	0.018	3.321	0.415
2000	380	512	0	0.007	3.098	0.502
2001	528	715	0	0.005	2.749	0.728
2002	893	1128	0	0.003	2.879	0.687
2003	920	1179	0	0.003	3.04	0.61
2004	1358	1708	0	0.002	2.786	0.713
2005	1623	2063	0	0.002	2.684	0.691
2006	1431	1755	0	0.002	2.773	0.643
2007	1188	1481	0	0.002	2.838	0.579
2008	1461	1760	0	0.002	2.793	0.679
2009	1248	1490	0	0.002	2.831	0.547
2010	978	1204	0	0.003	2.891	0.558
2011	1602	2565	0.172	0.002	2.911	0.491

Computational Biology. n:nodes, m: edges, $\langle C \rangle$: Clustering coefficient, $\langle p \rangle$: density, $\langle l \rangle$: Shortest average path length,NC: Network centralization
