# Supplementary material for: Mapping the Structure and Dynamics of Genomics-Related MeSH Terms Complex Networks
Source: PLoS One. 2014 Apr 3;9(4):e92639. doi: 10.1371/journal.pone.0092639 (PMC3974714; doi:10.1371/journal.pone.0092639)
Supplement: Table S7 — Data for the MeSH term Polymerase Chain Reac-tion networks for 23 years. First column contains the year, second column (n) is the number of nodes, third column (m) is the number of edges, fourth column () is the average clustering coefficient, fifth column () is the networks’ density, sixth column () is the shortest average path length, and seventh column (NC) is the network centralization. (PDF) [file pone.0092639.s007.pdf]

Table S7. Data for the MeSH term **Polymerase Chain Reaction** networks for 23 years.

**Years**	**n**	**m**	**⟨C⟩**	**⟨p⟩**	**⟨l⟩**	**NC**
1989	6	5	0	0.333	1.667	1
1990	13	12	0	0.0154	1.846	1
1991	35	34	0	0.057	1.943	1
1992	46	50	0	0.048	2.359	0.832
1993	61	63	0	0.034	2.487	0.809
1994	35	38	0	0.064	2.588	0.65
1995	49	48	0	0.041	1.959	1
1996	85	96	0	0.027	2.801	0.655
1997	57	60	0	0.038	3.013	0.516
1998	7	6	0	0.286	1.714	1
1999	70	73	0	0.03	2.865	0.655
2000	94	96	0	0.022	2.35	0.703
2001	158	183	0	0.015	2.825	0.637
2002	400	477	0	0.006	3.167	0.465
2003	370	424	0	0.006	3.056	0.561
2004	750	908	0.099	0.003	2.753	0.499
2005	1017	1236	0.103	0.002	2.766	0.584
2006	872	1030	0.068	0.003	2.665	0.684
2007	829	970	0	0.003	2.883	0.651
2008	993	1140	0	0.002	2.963	0.651
2009	1101	1280	0	0.002	2.796	0.716
2010	1200	1393	0	0.002	2.834	0.687
2011	1494	1885	0.102	0.002	2.809	0.493

Polymerase Chain Reaction. n:nodes, m: edges, $\langle C\rangle$: Clustering coefficient, $\langle p\rangle$: density, $\langle l\rangle$: Shortest average path length,NC: Network centralization
